# Tumor-associated microbiota in colorectal cancer with vascular tumor thrombus and neural invasion and association with clinical prognosis: Microbiota in colorectal cancer with vascular and neural invasion

**DOI:** 10.3724/abbs.2023255

**Published:** 2023-10-30

**Authors:** Mingjie Li, Min Jin, Lei Zhao, Dandan Yu, Yan Li, Linli Shi, Bin Zhou, Li Liu, Yinghao Cao, Kailin Cai, Jun Fan, Xiu Nie, Tao Zhang, Hongli Liu

**Affiliations:** 1 Cancer Center Union Hospital Tongji Medical College Huazhong University of Science and Technology Wuhan 430022 China; 2 Institute of Radiation. Oncology Union Hospital Tongji Medical College Huazhong University of Science and Technology Wuhan 430022 China; 3Department of Epidemiology and Biostatistics the Ministry of Education Key Lab of Environment and Health School of Public Health Tongji Medical College Huazhong University of Science and Technology Wuhan 430022 China; 4 Department of Gastrointestinal Surgery Union Hospital Tongji Medical College Huazhong University of Science and Technology Wuhan 430022 China; 5 Hubei Key Laboratory of Precision Radiation Oncology Wuhan 430022 China; 6 Department of Pathology Union Hospital Tongji Medical College Huazhong University of Science and Technology Wuhan 430022 China

**Keywords:** colorectal cancer, gut microbiota, 16S rRNA

## Abstract

Neural invasion (NI) and vascular tumor thrombus (VT) are associated with poor prognosis in patients with colorectal cancer (CRC). In this study, we apply 16S rRNA amplicon sequencing to tumor tissues and adjacent normal tissues in patients with CRC to determine the microbial differences. A discovery cohort, including 30 patients with NI, 23 with VT, and 35 with double-negative CRC tissue, is utilized. Then, we analyze the relationship between the specific bacterial taxa and indicators of different dimensions in separate cohorts. In the discovery cohort, the diversity and composition of the gut microbiome distinctly differ between the tumor and nontumor tissues in the NI and VT groups. A high abundance of *Cupriavidus* is found to be related to a short survival time of NI CRC, while *Herbaspirillum* is a potential microbial biomarker predicting the prognosis of patients with CRC with NI or VT. Moreover, the abundance of *Cupriavidus* or *Herbaspirillum* is associated with some clinical patient characteristics and prognosis, respectively. In conclusion, this study is the first to comprehensively elaborate the differences in the gut microbiota of patients with CRC with different invasion statuses and to prove the relationship between some gut microbiota and clinical patient characteristics.

## Introduction

Globally, colorectal cancer (CRC) is the third most common malignant cancer and the second most common cause of cancer-related deaths. Based on data from the Global Cancer Observatory, 1,880,725 new cases of CRC and 915,880 CRC-related deaths were recorded in 2020 [1]. Although the treatment strategies for CRC have improved in the past few years, the overall mortality remains high. In particular, distant invasion has long been a difficult challenge for clinicians because it is the most typical biological characteristic of malignant tumors. Approximately 50% of patients with CRC die of distant metastasis [2]. Therefore, finding specific biomarkers for invasive CRC, optimizing screening strategies and preventing metastasis are the focus of current research.

The vascular system is composed of three types of vessels: arterial, venous, and lymphatic. Invasion of the vascular system is widely regarded as the first stage in the formation of metastases. As an independent prognostic factor in patients with CRC, vascular tumor thrombus (VT), also known as vascular invasion, includes both lymphatic and blood vascular invasion. CRC spreads as invasive tumor cells that can either enter the lymphatic system and be delivered to regional lymph nodes or enter the blood vessels and spread throughout the body [3]. Histologically, vascular invasion is defined as the presence of tumor cells in the endothelium-lined luminal space or destruction of the vascular wall by tumor cells [4].

In contrast to direct spread and vascular metastasis, neural invasion (NI) invasively allows tumor cells to penetrate nerve fibers and disseminate along them. Fujita *et al*. [5] defined NI as tumor invasion of the Auerbach plexus in patients with CRC and identified it as a more important prognostic factor than vascular invasion. Accordingly, NI was added as a new criterion in the 7th edition of the tumor-node-metastasis classification of malignant tumors [6]. A previous study showed that the severity of NI has a significant impact on patient prognosis in colon cancer [7]. Moreover, a significant prognostic potential of NI and a higher incidence of local recurrence with escalating severity scores of NI was confirmed in patients with rectal cancer [8].

A large number of colonic microbiota studies suggest an association between CRC and infection with specific bacterial species, such as *Escherichia coli* [9]. The gut microbiota can also promote the metastasis of CRC through different mechanisms [10]. Although numerous studies on intestinal bacteria in patients with CRC have been undertaken, previous studies have not specifically addressed the probable changes across individuals with different invasion statuses [ 11– 14]. Limited data are available about the characteristics of the changes in the gut microbiota in aggressive CRC tissues.

In this study, we hypothesized that compared with double-negative (DN) CRC tissues, there may be variations in the abundance and composition of the intestinal microbiome of NI or VT CRC tissues. We verified whether there are differences in the microbiota status between tumor tissues and adjacent normal tissues, discovered the different microbiota of tumor tissues with different invasive states, and elucidated the relationship between specific microbiota and clinical characteristics of patients. Accordingly, we used 16S rRNA gene sequencing to evaluate tissue microbiota communities from patients with CRC who had NI or VT and determine whether the difference in the intestinal microbiome is connected to survival. Clarifying these potential distinctions is crucial for developing new screening and prevention strategies, as well as for promoting personalized treatment for CRC in the future.

## Materials and Methods

### Study participants and sample collection

A total of 329 Chinese patients (with 329 tumor tissue samples and 329 adjacent nontumor tissue samples) from Wuhan Union Hospital of Tongji Medical College, Huazhong University of Science and Technology, Central China, were enrolled in this study. Their data were collected from electronic medical records. Clinical data, including follow-up data and intestinal tissue samples of patients, were seriously evaluated, and the inclusion criteria for tissue sample selection were as follows: (1) patients with primary CRC who had undergone surgical therapy; (2) nonuse of antibiotics, nonsteroidal anti-inflammatory drugs, immunosuppressants, chemotherapy drugs, and other drugs affecting the gut microbiota 1 month before the collection of samples; and (3) availability of tumor and adjacent normal tissues. The exclusion criteria were as follows: history of alcoholism, smoking, and drug abuse, or unreasonable diet such as a high-fat diet that can disturb the gut microbiota composition [15].

Formalin-fixed, paraffin-embedded samples were used for the matched tumor and normal mucosal tissues. Five serial cuts sized 5 μm per sample were deposited in sterile containers and maintained at 23±2°C until 16S rRNA MiSeq was performed. All participants provided written informed consent before participation in the study. This experimental protocol was established according to the ethical guidelines of the Helsinki Declaration. This study was approved by the Ethics Committee of Tongji Medical College of Huazhong University of Science and Technology (approval No. 2014-041).

### DNA extraction, polymerase chain reaction amplification, and MiSeq

Genomic DNA was extracted from tissue samples using the Omega Mag-Bind Soil DNA Kit (Omega Bio-Tek, Norcross, USA). A NanoDrop (Thermo Fisher Scientific, Waltham, USA) was utilized for DNA quantification. The size of the DNA was checked via 0.8 agarose gel electrophoresis. All extracted DNA samples of the 329 patients were immediately stored at ‒80°C until analysis.

The extracted DNA was amplified using a series of primers targeting the V3-V4 variable region of the 16S rRNA gene, including the forward primer (5′-ACTCCTACGGGAGGCAGCA-3′) and the reverse primer (5′-GGACTACHVGGGTWTCTAAT-3′). The specific DNA barcode sequences for species identification were attached immediately after the adapter. Polymerase chain reaction (PCR) amplification was performed in a 25 μL reaction system containing 5 μL of 5× reaction buffer, 5 μL of 5× GC buffer, 2 μL of dNTPs (2.5 mM), 1 μL of forward primer (10 μM), 1 μL of reverse primer (10 μM), 8.75 μL of ddH _2_O, 0.25 μL of Q5 DNA polymerase (M0491L; NEB), and 2 μL of DNA template. PCR was then performed using an Applied Biosystems 2720 PCR instrument (ABI, Foster City, USA) under the following conditions: 2 min at 98°C; 30 cycles of 15 s at 98°C, 30 s at 55°C, and 30 s at 72°C; and final extension for 5 min at 72°C.

Agarose gel electrophoresis was performed twice to detect the PCR products. Amplicons were purified again and quantified using the Quant-iT PicoGreen dsDNA Assay Kit (Thermo Fisher Scientific) and an FLx800 microplate reader (Biotek, Winooski, USA). The DNA library was constructed using the TruSeq Nano DNA LT Library Prep Kit (Illumina, San Diego, USA), and sequencing was conducted using Illlumina MiSeq platform with MiSeq Reagent Kit v3 by Bioyi Biotechnology (Wuhan, China).

### 16S rRNA data analysis

The raw reads were quality filtered using QIIME2 (Quantitative Insights Into Microbial Ecology, v2019.4, https://qiime2.org/). Error tags, chimera, and low-quality sequences were all removed from the analysis. The clean data were clustered into operational taxonomic units (OTUs) at 97% sequence similarity using USEARCH and the UPARSE-OTU algorithm [16]. To remove sample heterogeneity before analysis, we performed rarefaction on the OTU table with QIIME2. Rarefaction allows users to standardize the data obtained from samples with different sequencing efforts and to compare the OTU richness of the samples using this standardized platform. The ecological approach of rarefaction is to randomly sample the same number of OTUs from each sample to predict the observed OTUs and their relative abundance of each sample at the sequencing depth and use these data to compare the communities at a given level of sampling effort. We selected the sequence with the highest abundance in each OTU as the representative sequence of each OTU. Using QIIME2 and the Greengenes database (release 13.8, http://greengenes.secondgenome.com/), the representative sequence and the corresponding abundance in the sample were taxonomically identified, which could be considered a taxon [17]. The alpha and beta diversities, including rarefaction curve, microbial diversity presented by Chao1, observed species and Good’s coverage diversity indices, permutational multivariate analysis of variance (PERMANOVA) according to Jaccard distance, principal coordinate analysis (PCoA) conducted by unweighted UniFrac distances, nonmetric multidimensional scaling (NMDS) and profiles of the microbial compositions, were calculated with QIIME2 and R according to the relative abundance of OTUs [18]. We also used an OTU abundance table to create Venn diagrams and counted the number of members of each set according to their presence in each group using R software, which represented the number of OTUs unique to each group or common among the groups.

The different taxonomies of the groups were identified using the linear discriminant analysis (LDA) effect size (LEfSe), and metabolic pathway prediction was performed by PICRUSt2 (Phylogenetic Investigation of Communities by Reconstruction of Unobserved States, v2.5.0, https://github.com/picrust/picrust2) [ 19, 20]. The LEfSe analysis results are shown as a histogram of the LDA value distribution of the significantly different species, which was used to reveal the significantly enriched species and their importance in each group. LEfSe places more emphasis on searching for stable differential species between groups; that is, only the differential species whose difference in abundance between two groups exceeds the LDA threshold can be considered biomarkers. In general, the biomarker species with significant differences among groups can be found according to the highest relative abundance, LDA score and *P* value of each taxon in the comparison. One of its characteristics is that it can not only analyze the difference in community composition in different sample groups but also select the indicator microbial groups that have obvious performance in different subgroups. PICRUSt2 can generally predict metabolic pathways in multiple functional databases, and we used MetaCyc ( https://metacyc.org/) as the reference database for this study. Based on these data, species with significantly different abundances in the different groups were identified as biomarkers, and the functional potential of bacterial communities could be predicted.

### Grouping strategy

**Figure 1:**
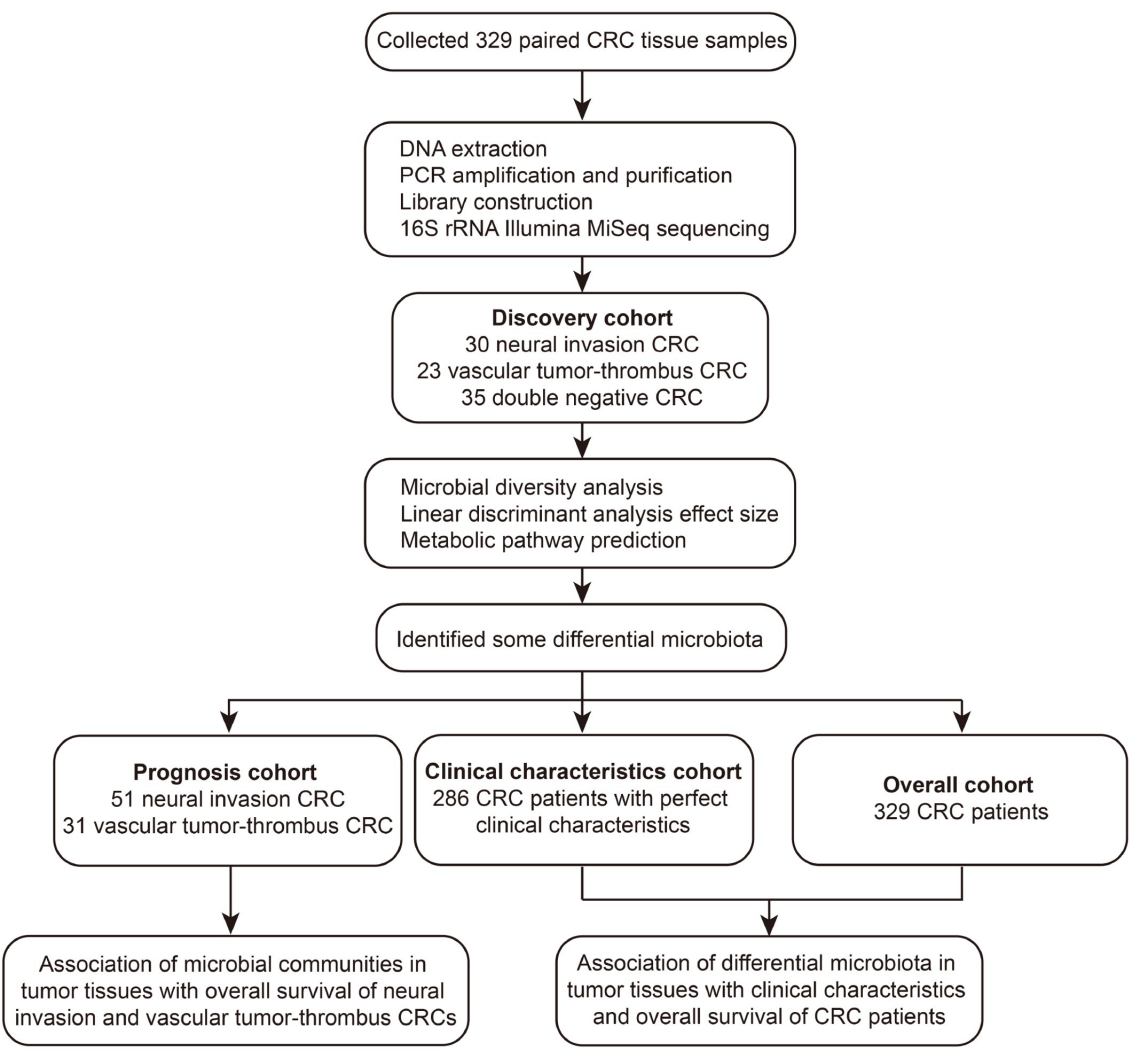
Figure 1 Study design and flow diagram

Figure 1 provides the cohort selection flowchart. In this study, a total of 329 CRC-matched tumor tissues and adjacent nontumor tissues comprised the overall cohort collected from Chinese patients. The above-indicated analysis was performed for all samples, and the annotation of all taxa and their abundance in all tissues, which were marked with the corresponding patient number, were obtained. After a strict pathological diagnosis, 30 paired NI CRC tissue samples (group NI), 23 paired VT CRC tissue samples (group VT), and 35 paired DN CRC tissue samples without NI and VT (group DN) were included as the discovery cohort. Supplementary Figure S1 shows the definition of NI or VT on pathological sections. Based on the clinical data of the patients and the abundance of various microorganisms detected using QIIME2 in the tumor tissues, we selected all patients with VT or NI (31 and 51 patients, respectively) from the overall cohort to form a prognosis cohort and explored the relationship between high/low abundance of the microbiota and the prognosis of patients with the corresponding invasion status. Subsequently, we selected some microbiota found in the previous analysis and explored the association of their respective abundance in the tumor tissues of 286 patients as an expanded cohort. Finally, we further analyzed the relationship between the abundance of the microbiota and the survival outcomes of the 329 patients.

### Statistical analysis

One-way analysis of variance and Chi-square test were used to compare demographics and tumor characteristics among patients with CRC with different invasive statuses in the discovery cohort. The results of 16S rRNA data analysis, including the alpha and beta diversities, were tested using QIIME2 and R. The post hoc tests were applied to evaluate the differences between tumor/nontumor tissues among the three groups of the discovery cohort (Dunnett test for the alpha diversity and Benjamini-Hochberg method for the beta diversity analysis and LEfSe). The enrichment degree of differential microbiota was categorized into “high” and “low” using the median value obtained from the 16S rRNA MiSeq sequencing as the cut-off point, while samples with undetectable microbiota expression were categorized as “negative”. Chi-square test, Fisher’s exact test, and *t*-test were used to compare the relationship between the abundance of selected microbiota and tumor characteristics. The Kaplan-Meier method was used to analyze the relationship between the abundances of microbiota and overall survival (OS) in the patients with CRC using R. All tests performed were two sided, and *P* or *q* values of <0.05 were considered statistically significant.

## Results

### Study design and patient demographic characteristics

There were a total of 329 CRC-matched tumor tissues and adjacent nontumor tissues collected from Chinese patients. After extracting DNA and detecting the enriched microbiota of all tissue samples by 16S rRNA Illumina MiSeq sequencing, we performed microbial diversity analysis, LEfSe and metabolic pathway prediction in the discovery cohort which included 30 neural invasion patients with CRC (group NI), 23 vascular tumor-thrombus patients with CRC (group VT) and 35 double-negative patients with CRC (group DN). Microbial community analysis was performed among the tumor tissues as well as among the nontumor tissues so that we could observe the differences in the composition and structure of microorganisms clearly in tumor or nontumor tissues with different invasive states and identified differential microbiota in the three groups. Then, patients in the prognosis cohort were divided into two groups according to the abundance of the most abundant genera, and the relationship between the abundance of several microbiota and the prognosis of patients with invasive status was discussed. Finally, we analyzed the correlation between the abundance of specific microbiota and the clinical characteristics of 286 patients in the clinical characteristics cohort and illustrated the association of the abundance of specific microbiota in tumor tissues with the overall survival time of patients in the overall cohort ( Figure 1). The clinical characteristics of the patients in the discovery cohort are shown in Supplementary Table S1. These data indicated that there were no significant differences in age, gender, tumor diameter, location or differentiation among the three groups (all *P*>0.05).

### Estimation of sequencing depth

**Figure 2:**
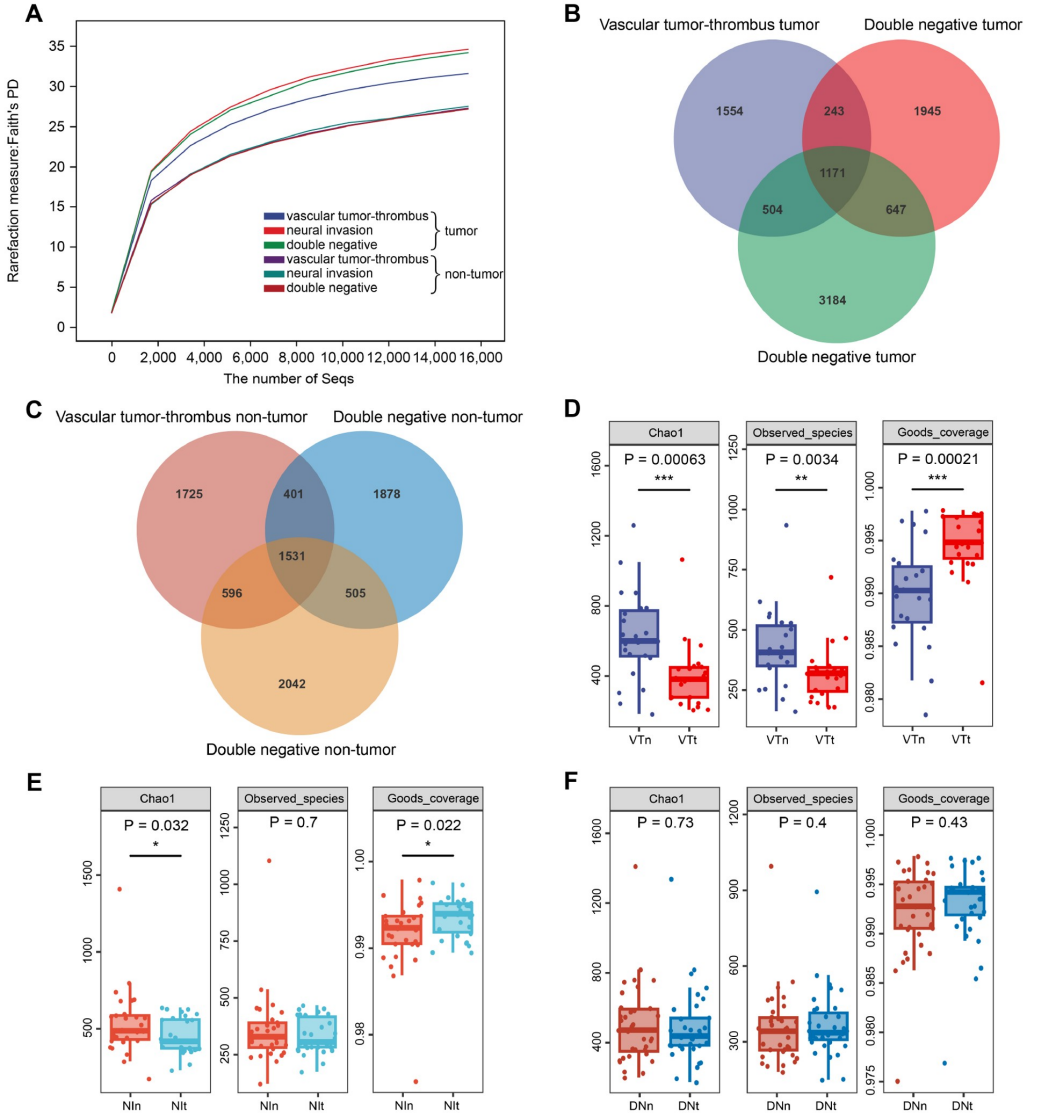
Figure 2 Microbial community richness among the three groups and alpha diversity between tumor and nontumor tissues in each group (A) Rarefaction curves between the number of sequences and estimated richness by Faith’s PD. The sequencing depth basically approached saturation in all samples. (B–C) Venn diagrams. The Venn diagrams represent the shared and unique taxa among the different tissues. (D–F) Microbial alpha diversity, as estimated by the Chao1 index, observed species index and Good’s coverage index. Dunnett’s test was used as the post hoc test. CRC, colorectal cancer; DNn, double-negative nontumor tissues; DNt, double-negative tumor tissues; NIn, neural invasion nontumor tissues; NIt, neural invasion tumor tissues; VTn, vascular tumor-thrombus nontumor tissues; VTt, vascular tumor-thrombus tumor tissues. *P<0.05, **P<0.01, ***P<0.001.

A data set of 203,358 high-quality sequences was obtained from 88 pairs of CRC tissue samples. The median length of each sample was 430 bp. Rarefaction curves based on Faith’s PD showed that the sequencing depth of all samples was close to saturation, indicating that the sequencing results are enough to reflect the diversity contained in the current samples ( Figure 2A). Among them, the genetic diversity of tumor tissues is higher than that of nontumor tissues. On the one hand, Venn diagrams illustrating the relationship among the groups showed that compared with DN tumor tissues, 1554 of 9248 OTUs were unique in VT tumor tissues, and 1945 OTUs were unique in NI tumor tissues ( Figure 2B). On the other hand, a Venn diagram also showed that only 1725 of 8678 OTUs were unique in VT nontumor tissues, and 1878 OTUs were unique in NI nontumor tissues ( Figure 2C). These data suggested that patients with CRC with different metastasis statuses have the possibility of containing distinctive microbial communities.

### Gut microbial alpha diversity

The alpha diversity indices of Chao1, observed species and Good’s coverage, which mainly indicate the species richness in the sample, were analyzed by using QIIME2 to estimate the richness and diversity of the microbial community in these samples. There were significant differences between the VT tumor tissues and adjacent nontumor tissues in the Chao1, observed species and Good’s coverage indices ( *P*=0.00063, 0.0034, and 0.00021, respectively) ( Figure 2D). The Chao1 and Good’s coverage indices also significantly differed between the NI tumor tissues and adjacent nontumor tissues ( *P*=0.032 and 0.022, respectively) but not for the observed species index ( *P*=0.7) ( Figure 2E). However, there were no significant differences found in these indices between the DN tumor tissues and adjacent nontumor tissues ( *P*=0.73, 0.4, and 0.43, respectively) ( Figure 2F). These results indicate that the nontumor samples have a greater species richness than tumor samples with VT and NI.

**Figure 3:**
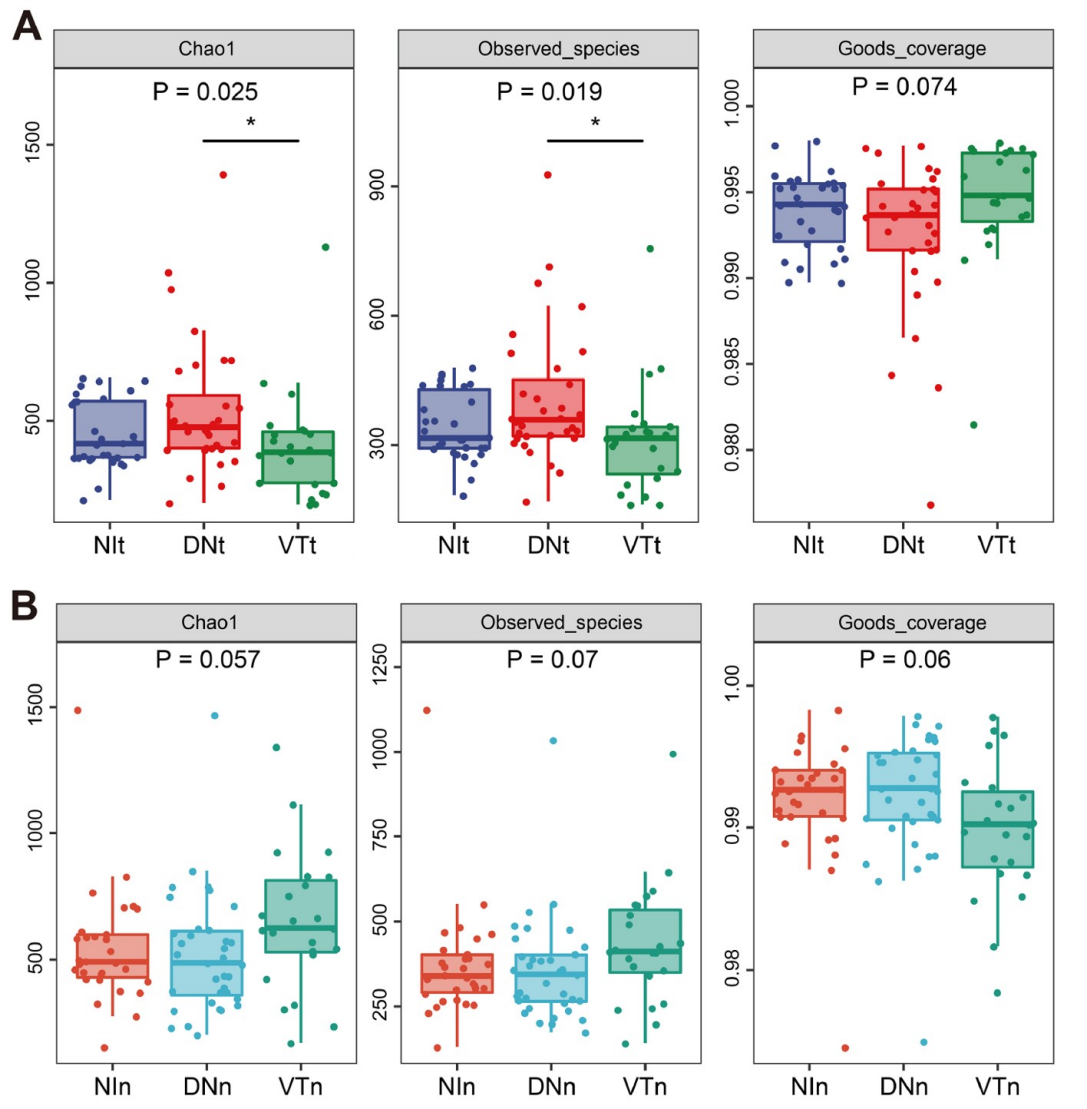
Figure 3 Microbial community richness and diversity between the tumor tissues or nontumor tissues of different groups (A,B) Microbial alpha diversity, as estimated by the Chao1 index, observed species index and Good’s coverage index. Dunnett’s test was used as the post hoc test. * P<0.05.

The Chao1 and observed species indices also significantly differed between the VT and DN tumor tissues ( *P*=0.025 and 0.019, respectively), except in the nontumor tissues. ( Figure 3A,B). Moreover, there were no significant differences between the NI and DN tissues or between the NI and VT tissues (all *P*>0.05) ( Figure 3A,B). These data indicated that there is a difference in the alpha diversity between the tumor tissues of the VT and DN groups but not between the nontumor tissues.

### Composition of the microbial community

The variations in the microbial compositions between paired samples from the three groups were determined using PERMANOVA to measure beta diversity. The neural invasion, vascular tumor-thrombus and double negative tumor tissues and their adjacent nontumor tissues had a statistically significant difference in beta diversity, according to Jaccard distance ( *P*=0.001, Table 1). However, the Jaccard distance did not significantly differ between the VT and DN tissues or between the NI and DN tissues (all *P*>0.05, Table 1) but did differ between the VT and NI tumor tissues ( *P*=0.016 and *q*=0.048, Table 1).

**Table:** **Table 1** Beta diversity assessed by Jaccard distances

Model	*F*	*R* ^2^	*P*-value	*q*-value
Tumor vs Non-tumor (vascular tumor-thrombus CRC)	2.891	0.062	0.000***	0.000 ^###^
Tumor vs Non-tumor (neural invasion CRC)	2.892	0.047	0.000***	0.000 ^###^
Tumor vs Non-tumor (double negative CRC)	2.617	0.037	0.000***	0.000 ^###^
Vascular tumor-thrombus vs Double negative (tumor)	1.132	0.020	0.193	0.290
Vascular tumor-thrombus vs. Double negative (non-tumor)	1.121	0.020	0.212	0.636
Neural invasion vs. Double negative (tumor)	1.017	0.017	0.362	0.362
Neural invasion vs Double negative (non-tumor)	0.886	0.014	0.636	0.636
Vascular tumor-thrombus vs Neural invasion (tumor)	1.436	0.027	0.016*	0.048 ^#^
Vascular tumor-thrombus vs. Neural invasion (non-tumor)	0.958	0.018	0.477	0.636

The *q*-value was determined with Benjamini-Hochberg method. CRC, colorectal cancer. * *P*<0.05, *** *P*<0.001; ^#^ *q*<0.05, ^###^ *q*<0.001.

The PCoA analysis based on the unweighted UniFrac distance matrix also proved that significant clustering was shaped in the tumor tissues of the three groups, which was distinguished from the nontumor tissues ( Supplementary Figure S2A–C). We intuitively discovered the most different microbial species between the tumor and nontumor tissues using LEfSe ( Supplementary Figure S2D-F). When the LDA threshold was 4.0, p_Thermi, c_Deinococci, o_Thermales, f_Thermaceae, f_Oxalobacteraceae, g_Thermus and g_Cupriavidus were enriched in tumor tissues. Simultaneously, p_Proteobacteria c_Alphaproteobacteria, o_Sphingomonadales and f_Sphingomonadaceae were enriched in the nontumor tissues compared with those in all tumor tissues. More interestingly, f_Pseudomonadaceae and g_Pseudomonas were highly enriched in the nontumor tissues of the VT and DN groups but not in the NI group.

Beta diversity analysis between the three groups was also evaluated through PCoA based on the unweighted UniFrac distance matrix and NMDS ( Supplementary Figure S3). Similar to the alpha diversity analysis results, no significant clustering was observed between the NI and DN groups. Although there were differences in the alpha diversity, no significant clustering was shaped between the VT and DN groups.

**Figure 4:**
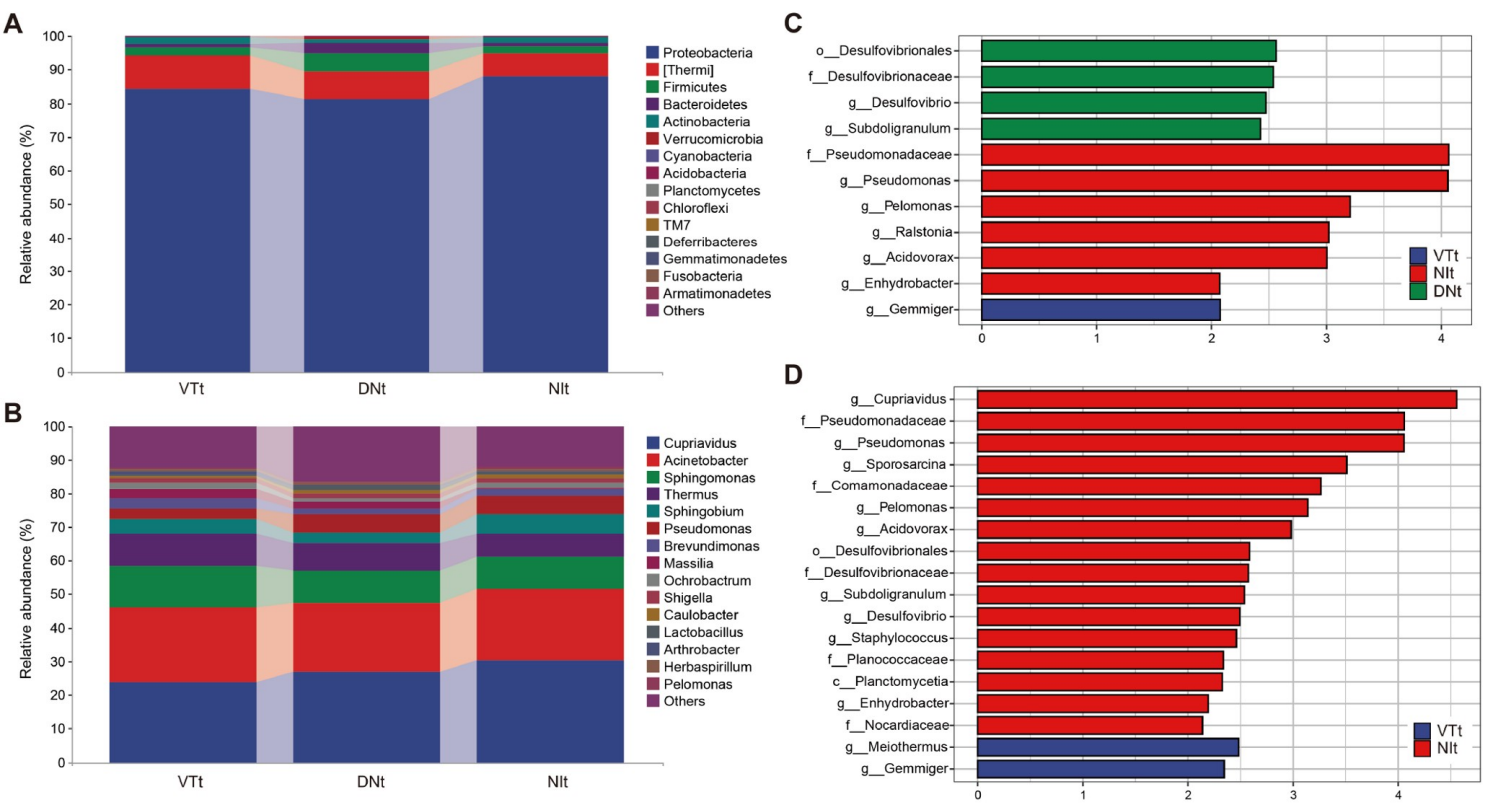
Figure 4 Profiles of the microbial compositions and LEfSe analysis among the three groups Microbiota compositions of gut microbiota in the tumor tissues at the phylum level (A) and genus level (B). (C,D) LEfSe analysis. The current LDA threshold is 2.0. The significantly differentially abundant bacteria are displayed in the histogram. The Benjamini-Hochberg method was used as the post hoc test. LDA, linear discriminant analysis; LEfSe, linear discriminant analysis effect size.

At the phylum level, the five most fundamental phyla in the 88 CRC tumor tissue samples were *Proteobacteria*, *Thermi*, *Firmicutes*, *Bacteroidetes* and *Actinobacteria*, accounting for 98% of the total community ( Figure 4A). According to the relative abundance of the phyla in each group, the general microbial community composition could be clearly seen. At the genus level, the four dominant genera in the tumor tissues were *Cupriavidus* , *Acinetobacter*, *Sphingobium* and *Thermus* ( Figure 4B). To further evaluate the microbial community differences between the tumor and nontumor tissues of the three groups, we conducted LEfSe. As expected, we did not find meaningful differences in the nontumor tissues; the different microbial species in the tumor tissues are shown in Figure 4C. When the LDA threshold was 2.0, the gut microbiota of the patients with NI was enriched with f_Pseudomonadaceae, g_Pseudomonas, g_Pelomonas, g_Ralstonia, g_Acidovorax and g_Enhydrobacter. In DN CRC tumor tissues, increased abundances of o_Desulfovibrionales, f_Desulfovibrionaceae, g_Desulfovibrio and g_Subdoligranulum were observed. Only g_Gemmiger was enriched in the patients with VT. The highest relative abundance, LDA score, *P* value and *q* value of each taxon among the three groups are also described in Supplementary Table S2. Furthermore, the different microbiota detected in LEfSe differed between the tumor tissues of the VT and NI groups, as also shown in the previous beta diversity analysis. A total of 18 enriched species were identified ( Figure 4D). g_Cupriavidus, f_Pseudomonadaceae and g_Pseudomonas were enriched in group NI, while g_Meiothermus and g_Gemmiger were enriched in group VT.

These data indicated that the heterogeneity of the gut microbiota in CRC tissues with different metastasis statuses.

### Metabolic pathway difference analysis between the tumor and nontumor tissues

We analyzed the differences in the metabolic pathways between the tumor and adjacent nontumor tissues using PICRUSt2 and MetaCyc in the discovery cohort. As shown in Supplementary Table S3, there were fewer differential metabolic pathways in the VT group than in the NI and DN groups, and there was no significantly downregulated metabolic pathway in the VT tumor tissues. Several metabolic pathways, such as mandelate degradation I, mandelate degradation to acetyl-CoA, and cytidine monophosphate-legionaminate biosynthesis I, were enriched in the tumor tissues of the NI and VT groups. Numerous metabolic pathways, including glyoxylate assimilation, the 3-hydroxypropanoate cycle, and the superpathway of the 3-hydroxypropanoate cycle, were significantly reduced in the tumor tissues of the DN and NI groups compared with those in the nontumor tissues. Moreover, seven metabolic pathways, including mevalonate pathway I, the superpathway of sulfolactate degradation, peptidoglycan biosynthesis, teichoic acid (poly-glycerol) biosynthesis, geranylgeranyl diphosphate biosynthesis and lactose, the superpathway of glycerol degradation to 1,3-propanediol, and galactose degradation, were highly increased in the tumor tissues of the three groups. However, we did not find significantly different metabolic pathways among the tumor tissues of the three groups, which indicates that there is no unique metabolic pathway in tumor tissues with different invasion statuses.

### Association of the microbial communities with outcomes and clinical characteristics

Based on the results of the three groups in the discovery cohort, we evaluated the association of high- or low-abundance microbiota in the tumor tissues and the survival of the patients with CRC with VT or NI in the prognosis cohort (31 and 51 patients, respectively). As shown in Table 2, we used the Kaplan–Meier method and found that among the 18 most highly abundant microbial communities at the genus level, the abundance of *Herbaspirillum* was significantly associated with the survival time of patients with CRC with VT or NI ( *P*=0.026 and 0.014, respectively). A high abundance of *Cupriavidus* in the NI CRC tissues was associated with a significantly shorter survival time ( *P*=0.019). Although *Pseudomonas*, *Pelomonas*, *Ralstonia* and *Acidovorax* were enriched in tumor tissues of the NI group, their abundance had no significant relationship with the prognosis of patients with neural invasion (all *P*>0.05).

**Table:** **Table 2** Association of microbial communities with overall survival of neural invasion and vascular tumor-thrombus CRCs

Microbial communities	OS in neural invasion patients with CRC (days)		OS in vascular tumor-thrombus patients with CRC (days)
	High	Low or negative	*P*-value		High	Low or negative	*P*-value
*Cupriavidus*	457	500	0.019*		443	582	0.546
*Acinetobacter*	448	510	0.558		459	574	0.193
*Sphingomonas*	423	536	0.145		567	459	0.497
*Thermus*	465	492	0.224		481	550	0.180
*Pseudomonas*	482	474	0.717		480	552	0.591
*Sphingobium*	493	463	0.528		586	439	0.486
*Brevundimonas*	505	450	0.547		491	536	0.413
*Massilia*	460	497	0.180		454	580	0.707
*Ochrobactrum*	433	526	0.150		395	642	0.491
*Shigella*	535	420	0.553		486	545	0.157
*Caulobacter*	523	432	0.125		464	568	0.683
*Lactobacillus*	542	413	0.113		496	534	0.544
*Arthrobacter*	457	501	0.710		552	474	0.871
*Herbaspirillum*	433	525	0.014*		485	546	0.026*
*Pelomonas*	436	522	0.134		492	539	0.733
*Aquabacterium*	446	511	0.652		520	509	0.609
*Acidovorax*	486	470	0.734		427	608	0.151
*Ralstonia*	442	517	0.691		497	533	0.204

We define the level of bacterial taxa (high and low or negative) based on the median abundance of microbiota in tumor tissues. OS data are shown as the mean. OS, overall survival. * *P* <0.05.

Considering that the small sample size in our previous analysis may not be widely representative, we verified the association of the status of the different taxa with the clinical characteristics of 286 patients with CRC. We selected four microbes mentioned in the previous experiment, including *Cupriavidus* , *Herbaspirillum*, *Thermus* and *Pseudomonas*. Interestingly, the status of *Cupriavidus* was associated with NI ( *P*=0.042, Table 3), pN stage ( *P*=0.006, Table 3) and clinical stage ( *P* =0.001, Table 3). An abundance of *Herbaspirillum* was also associated with tumor diameter ( *P* =0.001, Table 3), NI ( *P* =0.011, Table 3), pN stage ( *P* =0.013, Table 3), distant metastasis ( *P*=0.001, Table 3) and clinical stage ( *P*=0.001, Table 3). However, there was no significant relationship between the abundance of *Herbaspirillum* and the incidence of VT ( *P*=0.214, Table 3). Moreover, the abundance of *Thermus* was related to the pN stage ( *P*=0.013, Supplementary Table S4) and clinical stage ( *P*=0.043, Supplementary Table S4). Although *Pseudomonas* was highly enriched in the tumor tissues of patients with NI, its abundance was not significantly correlated with the clinical characteristics of the patients (all *P* >0.05, Supplementary Table S4).

**Table:** **Table 3** Characteristics according to the relative abundance of Cupriavidus and Herbaspirillum in CRC tissues

Characteristics	*Cupr*. high	*Cupr*. low	*P*-value	*Herb*. high	*Herb*. low/negative	*P*-value
	*n*=143 (%)	*n*=143 (%)		*n*=143 (%)	*n*=143 (%)	
Age (years)	57.94±12.93	55.07±13.17	0.064	57.52±12.99	55.49±13.20	0.190
Gender			0.233			0.811
Male	76 (53)	86 (60)		82 (57)	80 (56)	
Female	67 (47)	57 (40)		61 (43)	63 (44)	
Tumor diameter (cm)	4.51±2.24	4.86±2.24	0.190	4.26±2.08	5.11±2.33	0.000***
Neural invasion			0.042*			0.011*
Absent	90 (63)	106 (74)		88 (62)	108 (76)	
Present	53 (37)	37 (26)		55 (38)	35 (24)	
Vascular tumor-thrombus			0.890			0.214
Absent	109 (76)	108 (76)		104 (73)	113 (79)	
Present	34 (24)	35 (24)		39 (27)	30 (21)	
pT stage			0.657			1.000
T1+T2	10 (7)	12 (8)		11 (8)	11 (8)	
T3+T4	133 (93)	131 (92)		132 (92)	132 (92)	
pN stage			0.006**			0.013*
N0	63 (44)	86 (60)		64 (45)	85 (59)	
N1+N2	80 (56)	57 (40)		79 (55)	58 (41)	
Distant metastasis			0.227			0.000***
M0	112 (78)	120 (84)		105 (73)	127 (89)	
M1	31 (22)	23 (16)		38 (27)	16 (11)	
Clinical stage			0.000***			0.000***
1+2	50 (35)	77 (54)		48 (34)	79 (55)	
3+4	93 (65)	66 (46)		93 (65)	66 (46)	

The *P*-values for categorical and continuous variables were analyzed by Chi-square test and Fisher’s exact test, and continuous variables were analyzed by two independent-samples *t*-test. * *P*<0.05, ** *P*<0.01, *** *P*<0.001. *Cupr*., *Cupriavidus*; *Herb*., *Herbaspirillum*.

**Figure 5:**
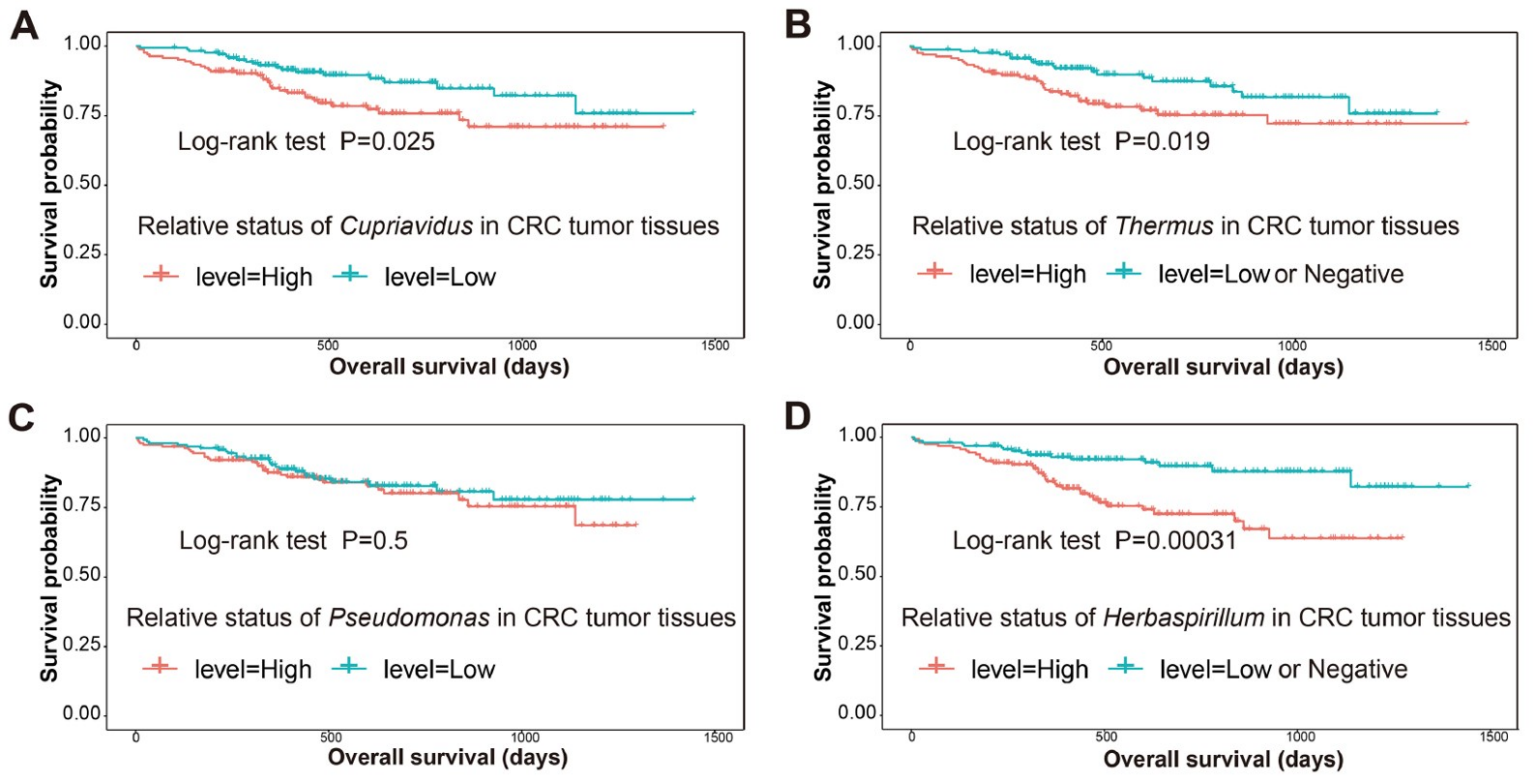
Figure 5 Association of four unique flora enriched in tumor tissues with the clinical overall survival of patents Kaplan–Meier curves for colorectal cancer overall survival according to the relative status of Cupriavidus in CRC tumor tissues (A), the relative status of Thermus in CRC tumor tissues (B), the relative status of Pseudomonas in CRC tumor tissues (C), and the relative status of Herbaspirillum in CRC tumor tissues (D). The level of microflora (high and low or negative) was defined based on the median abundance of microbiota in tumor tissues. The P value was calculated by the log-rank test. CRC, colorectal cancer.

Unfortunately, we did not see any significant differences in the prognostic follow-up data of the patients in the discovery cohort with a small sample size (all *P*>0.05, Supplementary Table S5). Then, we tested the association of the different microbiota with the outcomes of the overall cohort based on detailed follow-up records through Kaplan‒Meier analysis. The median survival time of the patients with CRC with highly abundant *Cupriavidus* was significantly shorter than that of the patients with scarce *Cupriavidus* ( *P* =0.025, Figure 5A). Meanwhile, there was a significant difference between *Thermus*-high patients and *Thermus*-low/negative patients ( *P*=0.019, Figure 5B). However, in patients with different abundances of *Pseudomonas* in tumor tissues, there was no significant difference ( *P*=0.5, Figure 5C). It was also observed that the difference in the survival time between *Herbaspirillum*-high and *Herbaspirillum*-low/negative patients was statistically significant ( *P*=0.00031, Figure 5D).

These data indicated that *Cupriavidus* and *Herbaspirillum* are the potential microbial biomarkers predicting the prognosis of patients with CRC with NI or VT, and the abundance of them are closely related to the diagnosis of CRC.

## Discussion

When compared to the gut microbiota of healthy people, the gut microbiota of people with CRC has a lower abundance of possibly protective taxa and a higher abundance of procarcinogenic taxa [21]. Riquelme *et al*. [22] found that a lower alpha diversity in the tumor microbiome was associated with short-term survival in patients with pancreatic adenocarcinoma. Among patients with gastric cancer, those with liver metastasis have a higher alpha diversity and worse prognosis [23]. Our study further illustrated that the total abundance of the gut microbiota in CRC tumor tissues with invasion is lower than that in adjacent nontumor tissues. Tumors with VT have lower species richness of the gut microbiota than tumors with DN tissue, while the beta diversity does not differ. In contrast, there was a difference in the beta diversity between the tumor tissues of the VT and NI groups but no difference in the alpha diversity. By using LEfSe, we also discovered that the gut microbiota of patients with NI was enriched with Pseudomonas, Pelomonas, Ralstonia, Acidovorax and Enhydrobacter at the genus level. Increased abundances of Desulfovibrio and Subdoligranulum were observed in DN CRC tumor tissues. Only Gemmiger was enriched in the patients with VT. Taken together, the effects of microbiota diversity on tumor growth and patient survival do not remain the same across different tumor types. However, our results are inconsistent with previous reports that *Proteobacteria* was enriched in tumor tissues and that tumor microbiomes showed a higher alpha diversity than did normal, patient-matched microbiomes [24]. These incompatible results may be attributed to the fact that patients come from different geographical environments and races and may have different dietary habits, which can affect the nutritional structure of the intestines [ 25, 26].

*Cupriavidus* is characterized as a gram-negative, motile, rod-shaped organism with oxidative metabolism and is found in both soil and clinical isolates, particularly in samples from debilitated patients [27]. Previous studies have shown that *Cupriavidus* is an organophosphorus pesticide-degrading microorganism that is also highly enriched in the tumor tissues of patients with bladder cancer [28]. In our study, LEfSe showed that *Cupriavidus* had a large amount of aggregation in the tumor tissues; we proved for the first time that there was a significant correlation between the abundance of *Cupriavidus* in the tumor tissues and the survival time of patients with CRC, especially those with NI. Although *Cupriavidus* was not enriched in the NI group, a high abundance of this genus was closely associated with the existence of adverse clinical characteristics, including NI and advanced clinical stage; this finding suggests that this genus plays an important role in the development of tumors, which may be an important focus for future prevention and treatment of NI CRCs. We speculate that the enzymes of this genus of bacteria may play a crucial role in tumor tissues because this genus has been demonstrated to complete a variety of chemical reactions *in vitro*, including Mn-oxidizing and producing chemicals from CO _2_ [29]; however, the specific mechanisms of interaction between this bacterium and CRC need to be confirmed in future studies.

*Thermus*, a bacterium belonging to the *Thermi* (also known as *Deinococcota* or *Deinococcus-Thermus*) phylum, is a genus of thermophilic bacteria [30]. It has been revealed that the genus *Thermus* is more abundant in tissues from patients with advanced stages of lung cancer or HPV 16 infection [ 31 , 32]. In this study, we found that *Thermus* was highly enriched in tumor tissues compared with nontumor tissues and was related to the survival time as well as some clinical characteristics of patients with CRC. Interestingly, in a previous study on chickens, *Thermus* was significantly abundant in low-body-weight chickens, which showed significantly upregulated inflammatory cytokines in the jejunum [33]. We speculate that the effect of *Thermus* in the human intestinal tract is also related to inflammatory responses, leading to damage of gut barrier integrity and thus affecting the nutrient absorption of the intestines. However, the role of this genus in the intestinal microbiota, especially in the formation and development of CRC, remains controversial. Although there was no significant relationship found between *Thermus* and the incidence of NI and VT in CRCs, the impact of this genus on the prognosis of patients requires further investigation.

Concurrently, we found that the abundance of *Herbaspirillum*, which is a bacterial taxon that was not enriched in the previous analysis, was associated with the survival time of the patients with CRC. As a gram-negative, strictly aerobic, curved or helical bacilli member of the *Betaproteobacteria* class, *Herbaspirillum* is a plant bacterium that is motile with polar flagella but was not previously considered a human pathogen [34]. With advancements in detection technology, *Herbaspirillum* has been reported as a new opportunistic pathogen in patients with cancer or those who have undergone hematopoietic stem cell transplant [35]. To our knowledge, this study is the first to elucidate that the massive accumulation of *Herbaspirillum* in the tumor tissues of the intestines is related to the tumor diameter, NI, distant metastasis, and clinical stage. However, a smaller tumor diameter and a higher incidence of NI were associated with a higher abundance of *Herbaspirillum*, indicating that this genus of bacteria contributes to the invasion rather than the growth of tumor tissues. A high abundance of *Herbaspirillum* was also strongly associated with poor prognosis in patients with VT but had no relationship with the incidence of VT, suggesting that *Herbaspirillum* infection is a risk factor leading to a short survival time in patients with VT. The virulence of *Herbaspirillum* may be related to flagella because flagellar movement could enhance the invasion of bacteria to the host, and the movement could avoid harmful environments [34]. Faoro *et al*. [36] also found that the clinical strains of *Herbaspirillum* acquired new genes and genomic islands, including a cluster of genes related to lipopolysaccharide (LPS) biosynthesis that may be involved in host interactions. Interestingly, the high enrichment of this genus in tumor tissues is consistent with previous findings that LPS is highly present in CRC tumor tissues and is known for its role in intestinal inflammation and CRC progression [37]. Furthermore, *Herbaspirillum* infection may be caused by ingestion of undercooked vegetables or contaminated water, as this species is a water- and soil-based organism [ 38 , 39]. Considering that most of our patients resided in places irrigated by rivers, we speculate that there was already a certain amount of *Herbaspirillum* in their intestines, which may explain why there was no enrichment of this genus in the previous LEfSe. Therefore, we believe that the condition of patients with CRC may be aggravated by severe *Herbaspirillum* infection, which can cause inflammatory reactions in tumor tissues through the biosynthesis of LPS and can also produce host interactions through flagellar movement. However, the detailed mechanism of this genus in the microenvironment of CRC tissues is still unknown, and relevant research needs to be continued in the future.

In previous studies, sulfide-producing pathways, cholesterol biosynthesis, and elements of central carbon metabolism were found to be closely related to CRC and gut microbiota [ 40, 41]. In this study, we also found a few metabolic pathways involved in the above biosynthesis, including the mevalonate pathway, superpathway of sulfolactate degradation and superpathway of glycerol degradation to 1,3-propanediol, which were enriched in the tumor tissues. Mevalonate pathway enzymes are related to the gut microbiota, and antibiotics can effectively inhibit their concentration, reducing systemic and intestinal inflammation [42]. Therefore, the microbiota within CRC tumor tissues can cause an inflammatory response, which can contribute to the formation of the tumor microenvironment. Moreover, 2,3-dihydroxypropane-1-sulfonate, one of the most abundant organic sulfur compounds in the biosphere, has been proven to be degraded by *Cupriavidus*, and its degradation products can impact human health [ 43, 44]. This may be one reason why the abundance of *Cupriavidus* in the tumor tissues was higher and correlated with the prognosis of the patients with CRC. Although the relationship between the metabolic pathways enriched in tumor tissues and the high abundance of bacteria found in this study is unknown, our findings provide a new basis for the investigation of CRC via metabolomics.

The strength of this study was that we collected a relatively large number of samples and integrated the basic information of tumor samples and patients. We also used follow-up survival data to analyze the microbial differences between the tumor tissues and adjacent nontumor tissues of patients with CRC with NI or VT for the first time and to explore the relationship between the clinical characteristics (including survival time) of the patients and the abundance of different microbiota. The sequencing data of this study have been uploaded to the SRA database with the accession numbers SRR12019460 continuity to SRR12020117 and SRR20281361 continuity to SRR20281373. Additional patient clinical information with sample identification and survival outcomes is presented in Supplementary Table S6.

This study also had several limitations. First, potential DNA contamination, which is the most serious problem, may exist in the samples we analyzed. In particular, a previous study listed *Cupriavidus* and *Herbaspirillum* as contaminant genera [45]. To be exact, this article is aimed at improper operation with samples containing a low microbial biomass, which is less than approximately 10 ^3^ to 10 ^4^ cells. Given our collection methods, careful histological review and samples containing relatively large microbiota, it is unlikely that the tissue samples from patients with CRC were contaminated. In addition, our validation study showed that phyla and genera were enriched in CRC tumor tissues, which is consistent with a previous study [46]. Second, we did not test additional microorganism specimens, such as saliva and feces, from the patients. Third, the patients were recruited from a single large-scale general hospital, and their information was evaluated retrospectively. Therefore, the geographical environment, dietary structure, and customs of patients may have impacted the results. However, considering the harm of NI and VT in human CRC and the complex composition of the gut microbiota, this retrospective study also provides several new results with important reference value. Prospective investigations are nevertheless needed in the future to verify the heterogeneity of the gut microbiota in patients with CRC with different metastasis statuses and the specific roles of the above-indicated genera in tumor tissues. Furthermore, the gut microbiota has also been used to investigate whether it is related to unresponsiveness to cancer treatments, such as immunotherapy, in patients with cancer [47]. These and other studies have shown single microorganisms, consortia of microbes, and microbial metabolites that are being identified to increase the effectiveness of cancer treatments. Aside from CRC prevention, diagnosis, and treatment, potentially feasible opportunities exist with the use of microbiota profiling information in gastrointestinal microorganism-targeted therapies to decrease the toxicities of CRC therapeutics. We believe that the new findings revealed in this study can lay a solid foundation for the implementation of cell or animal experiments to clarify the mechanism of the interaction between the gut microbiota and invasive CRC. Eventually, future work may establish new biomarkers to judge the prognosis of patients and provide new treatments based on the application of probiotic strains.

## Supporting information

125TabS1

## Supplementary Data

Supplementary data are available at *Acta Biochimica et Biophysica Sinica* online.
