# Supplementary material for: Tumor-associated microbiota in colorectal cancer with vascular tumor thrombus and neural invasion and association with clinical prognosis: Microbiota in colorectal cancer with vascular and neural invasion
Source: Acta Biochim Biophys Sin (Shanghai). 2023 Oct 30;56(3):366–78. doi: 10.3724/abbs.2023255 (PMC10984857; doi:10.3724/abbs.2023255)
Supplement: 125TabS1 [file 125TabS1.pdf]

Supplementary Table S1. Sequences of the primers used in PCR

Target	Sequence (5′→3′)	Product (bp)
GAPDH	F: TGTTCGTCATGGGTGTGAAC	154
	R: ATGGCATGGACTGTGGTCAT	
CLPP	F: TGGAGCAGACGGGTCG	125
	R: GAGGAGCTGTGCGATAACAA	
HSPA8	F: TCAGGTTTATGAAGGCGAGC	158
	R: TGTCCACAGCAGAGACATTG	
